# Identifying psychosocial problems, needs, and coping mechanisms of adolescent Syrian refugees in Jordan

**DOI:** 10.3389/fpsyt.2023.1184098

**Published:** 2023-06-22

**Authors:** Tariq N. Al-Shatanawi, Yousef Khader, Husam ALSalamat, Lourance Al Hadid, Alaa Jarboua, Basil Amarneh, Osama Alkouri, Mahmoud A. Alfaqih, Nasr Alrabadi

**Affiliations:** ^1^Department of Public Health and Community Medicine, Faculty of Medicine, Al-Balqa Applied University, Al-Salt, Jordan; ^2^Department of Public Health and Community Medicine, Faculty of Medicine, Jordan University of Science and Technology, Irbid, Jordan; ^3^Department of Biopharmaceutics and Clinical Pharmacy, School of Pharmacy, The University of Jordan, Amman, Jordan; ^4^Faculty of Nursing, Al-Balqa Applied University, Al-Salt, Jordan; ^5^Department of Legal Medicine, Toxicology and Forensic Medicine, Faculty of Medicine, Jordan University of Science and Technology, Irbid, Jordan; ^6^Department of Community and Mental Health Nursing, Faculty of Nursing, Jordan University of Science and Technology, Irbid, Jordan; ^7^Faculty of Nursing, Yarmouk University, Irbid, Jordan; ^8^Department of Physiology and Biochemistry, Faculty of Medicine, Jordan University of Science and Technology, Irbid, Jordan; ^9^Department of Biochemistry, College of Medicine and Medical Sciences, Arabian Gulf University, Manama, Bahrain; ^10^Department of Pharmacology, Faculty of Medicine, Jordan University of Science and Technology, Irbid, Jordan

**Keywords:** psychosocial problems, needs, Syrian refugees, adolescents, coping mechanism

## Abstract

**Background:**

Refugees who have fled war zones are at a heightened risk of psychosocial problems that can impact their ability to function in day-to-day life and place a significant burden on the family structure. This study aimed to assess the psychosocial problems and needs and coping mechanisms of Adolescent Syrian refugees in Jordan.

**Methods:**

Between October and December 2018, we conducted a qualitative study using semi-structured interviews with a sample of key and individual informants. Our sample included 20 primary healthcare professionals, 20 schoolteachers, 20 Syrian parents, and 20 adolescents aged 12–17 years. All interviews were transcribed verbatim, and we utilized thematic analysis to group, categorize, and analyze the original Arabic language transcripts. To ensure thorough analysis, we adopted a bottom-up inductive approach that covered the six-phase iterative process proposed by Braun and Clarke.

**Results:**

The main psychosocial problems encountered by Syrian adolescents included stress, depression, loneliness, lack of a sense of security, isolation, aggressiveness, fear of war, and family disintegration. Almost all schoolteachers reported that they observed that Jordanian adolescents are more settled, self-confident, and financially stable relative to their Syrian peers. The Jordanian government and community were praised for their support, including education, recreational centers, health services, and awareness campaigns. Going to school, praying and reading the Holy Quran, listening to music, and talking to friends and engaging with them were reported as the main coping mechanisms. The majority of respondents said that more services are still needed for adolescents, including more places for entertainment, psychosocial support and psychological counseling, medical care, job creation, and providing health insurance.

**Conclusion:**

Syrian refugees are aware of the psychological aspects of their situation, but they are not always able to access clinic-based humanitarian assistance for mental health and psychosocial support. Stakeholders must interact with refugees to learn about their needs and develop services that are appropriate for their culture.

## Introduction

Since the start of the conflict in Syria in 2011, 27% of all refugees worldwide are Syrians with about 6.1 million individuals displaced within Syria, and about 6.8 million refugees have fled to 129 countries, including Jordan which hosts around 673,00 Syrian refugees (1). More than 50% of Syrian refugees are children under the age of 18 years (2).

In the past, research on refugees has largely focused on establishing correlations between exposure to immigration and negative mental health outcomes. However, in recent years, there has been a growing body of research that examines the impact of chronic daily stressors that arise during the resettlement process (3). The need for psychosocial care is closely linked with the migration movement, particularly for individuals who have experienced the loss of family members, close friends, shelter, home, or neighborhood. Migration can be a traumatic experience, and the loss of one’s familiar environment and support systems can exacerbate existing psychosocial issues or create new ones. In addition, refugees may face discrimination, marginalization, and social exclusion in their host communities, further affecting their mental health and well-being.

Several studies indicated that children and adolescents are the most severely affected by psychosocial problems like post-traumatic stress syndrome, panic attacks, sleep disorders, depression, suicidality, and others (4), all of which are particularly significant among refugees (5–7). Youth mental health may also be impacted by broader socioeconomic variables specific to each country of resettlement and regulations regarding the detention of refugees and access to medical care (8). Failure to cope with these problems may result in difficulties in sleeping, mood swings, isolation, grief, and lower activity (9, 10).

Syrian refugees, who fled their country, are particularly vulnerable to psychosocial problems (11–13). Not only can the crisis in Syria exacerbate pre-existing mental disorders within the refugees, but it could also trigger the surfacing of multiple psychosocial problems (14, 15). Previous studies have investigated the psychosocial problems and needs among Syrian refugee adolescents (16–29). However, few studies looked at Syrian adolescents in Jordan (30–37). Information on psychosocial problems and needs is needed to inform public policy and to develop adapted psychosocial services and programs. Moreover, collective analysis of the problem from the view of the adolescents themselves along with the view of their parents, educators, and healthcare professionals is warranted for in-depth understanding. Therefore, this study was conducted to assess the psychosocial problems and needs, and coping mechanisms of Adolescent Syrian refugees in Jordan after 8 years of the Syrian conflict.

## Methods

### Design and population

A qualitative study using semi-structured interviews with a sample of key and individual informants in four major cities in Jordan; Ramtha, Sahab, Mafraq, and Zarqa was conducted. The investigators chose these cities because they have the greatest number of Syrian refugees among other Jordanian cities. For key informant interviews, five primary healthcare centers and five schools were selected from each city. One health professional was nominated by the director of each health center and one schoolteacher was nominated by the principal of each school to be interviewed by the investigators. In total, the interviews were conducted with five primary healthcare professionals and five schoolteachers from each city (*n* = 20 each). In addition, individual interviews were conducted with five Syrian parents and five Syrian adolescents aged 12–17 years from each city. The parents and adolescents were suggested by the interviewed schoolteachers. Inclusion criteria for the adolescent included having no previously diagnosed or known psychological problems or any learning difficulties.

### Data collection

The interviews were conducted from October to December 2018. All interviews were conducted in quiet surroundings at a place described as acceptable by the informants. All interviews were conducted in the Arabic language, and medical jargon was avoided where possible, except in the case of the interviews with primary health care professionals. Two investigators led and moderated the interviewees. The moderator (female) guided the discussion based on the interview guide (Supplementary Table 1) that included 5 to 11 open-ended questions, depending on the type of respondents. The second investigator (male) was responsible for taking notes. We audio-recorded the interviews after obtaining approval from the interviewees. The interviews with adolescents were conducted in the absence of their parents.

The development of the interview guide was guided by the published literature review (16–28, 30–37) on the topic and by consultation with a group of experts that included a psychologist, a sociologist, a family physician, and two schoolteachers. Each consultant was approached by the investigators, provided with the developed tool and requested to provide feedback on the clarity, comprehensiveness, and appropriateness of the questions. All consultants confirmed that the questions were clear, comprehensible, and appropriate to the topic (face validity).

Adolescents were asked to list the types of psychosocial problems they encountered, what they did to cope with them, and where they went for help. They were also asked to state which psychosocial problems were critical and most important, and the impact of these problems on daily functioning. Questions for health care professionals and schoolteachers focused on the nature of the distress experienced by adolescents, and how assistance and support were being sought by adolescents. They were asked about their experiences in dealing with psychosocial problems affecting adolescent Syrians in Jordan. In addition, they were asked whether they noted any gender differences in these problems and in coping with the crisis, the effects of these problems on adolescents and what could be done to overcome these problems, the required health services, psychosocial support, and other kinds of assistance. Syrian parents were asked about the psychosocial problems of their children and the sources of these problems, the types of violence experienced by their children, how the problems had influenced their children’s behavior and feelings, and the source(s) of tension between Jordanians and Syrian refugees. The interview with each primary healthcare professional, schoolteacher, and parent lasted on average 45 min (range: 35–65 min), while that for adolescents lasted 55 min on average (Range: 50–75 min).

### Ethical considerations

Ethical approval was obtained from the Institutional Research Committee of Jordan University of Science and Technology. All informants signed a consent form before recruitment after full disclosure of the study’s aims and process. The participants were informed about the purpose of the study and the expected benefits of this research. They were informed that this research is associated with minimal risk. The interview moderator ensured privacy during the interview. The researchers informed all informants that their participation in this study is voluntary and that all information they provide shall be kept confidential and interview records will be put in a locked cabinet with only limited access by the project team. Similarly, the interviewers emphasized that any stress the informants can experience or a sense of discomfort with the proceedings of the interview, they can withdraw from the study. All study procedures were consistent with the guidelines of the Helsinki Declaration and its amendments.

### Data analysis

All interviews were transcribed verbatim. The thematic analysis was used to group, categorize, and analyze the original Arabic language transcripts, which would lead to the conservation of the credibility of the findings. Analysis of the transcribed information was done manually adopting the process of coding and category generation and theme recognition. The thematic analysis adopted a bottom-up inductive approach which covers the six-phase iterative process as proposed by Braun and Clarke (38) which includes (1) understanding the data; (2) producing first codes (3) finding topics; (4) analyzing themes; (5) identifying themes; and (6) writing the report. The thematic analysis consisted of a full reading of the whole transcripts by two independent researchers from the project team, followed by highlighting and coding important sentences into different categories several times until themes and sub-themes were identified and agreed upon. Translation of themes, as well as quotes into the English language, was performed by two bilingual experts. Table 1 summarizes the main outcome of the thematic analysis and the Supplementary Table 2 shows the qualitative analysis outputs.

**Table 1:** Thematic analysis from key informants and parents.

Themes/categories	Response interpretations
Psychosocial problems Syrian adolescents encounter in Jordan	Stress
	Depression
	Isolation
	Aggressiveness
	Family disintegration
	Racial discrimination
	Violence
	Poor educational attainment
	Bed-wetting
	Poverty
	Early marriage
Psychosocial problems: differences between Jordanian and Syrian adolescents	Jordanian adolescents were safer, financially stable, and socially supported than Syrian adolescents
	Jordanian adolescents were not subjected to war-related atrocities similar to Syrian ones, but they experienced family-related problems
	Syrian adolescents were more emotionally intelligent and aware than Jordanian adolescents
Psychosocial problems: gender differences	Adolescent Syrian females were at a bigger disadvantage than their male counterparts
Types of bullying common among Syrian and Jordanian adolescent	Verbal and physical forms of bullying were the most common among both communities
Coping mechanisms used to overcoming problems	Activities supporting the integration of the two communities
	Awareness and education campaigns
	Psychosocial support and counseling
	Financial support
	Endeavors to eliminate racial discrimination between Jordanians and Syrians
	Seeking help from security agencies
	Recreational activities
Unmet psychosocial and other needs of Syrian adolescents	Free and specialized health services
	Health education
	Safe social environment
	Social support
The perceived role of institutions and organizations in help of adolescents	Some, not all, of the Syrian parents reported that some institutions introduced help and support for their children

### Trustworthiness

As part of considering the rigor of the qualitative data collected in this study, the member-checking method has been adopted with each informant. This process was used to validate informants’ narrative responses and their reflections on the experience they had. The validity of the data collection and analysis was enhanced by involving two researchers in reading, rereading, and categorizing the data. To maximize rapport during data collection, a friendly atmosphere was ensured and each informant received a full disclosure about the study to emphasize a risk-free context. Likewise, a third researcher was involved in the analysis process to provide coding, which contributed to the credibility of the data analysis. The transferability of the study findings was taken into account by a detailed account of the study’s conceptual and implemented steps, the settings, informants, data analysis, and the results that reflect what the informants said.

## Results

### Participants’ characteristics

The researchers interviewed 80 participants (20 healthcare professionals, 20 schoolteachers, 20 Syrian parents, and 20 adolescents). The characteristics of the participants are shown in Table 2.

**Table 2:** The demographic characteristics of study participants (*n* = 80*).

		Primary healthcare professionals	Schoolteachers	Syrian parents	Syrian adolescents
		*n* (%)	*n* (%)	*n* (%)	*n* (%)
Age in years	Range	30–52	28–41	30–62	12–17
	Mean (SD)	35.1 (3.3)	34.9 (3.3)	44.9 (3.3)	15.9 (2.0)
Gender	Female	9 (45.0)	8 (40.0)	8 (40.0)	10 (50.0)
	Male	11 (55.0)	12 (60.0)	12 (60.0)	10 (50.0)

* Each category of participants includes 20 informants. SD: standard deviation; n: number.

### Psychosocial problems among Syrian adolescents

Based on their experiences and practices with adolescents, most healthcare professionals, schoolteachers, and Syrian parents reported that stress, depression, loneliness, lack of a sense of security, isolation, aggressiveness, fear of war, and family disintegration were among the main psychosocial concerns that Syrian adolescents encountered. The majority of adolescents reported that they had experienced such problems. Loss of school years, poor educational attainment, and difficulties in coping with the refugees or the new environment were reported by almost two thirds of schoolteachers, Syrian parents, and adolescents. Almost 60% of Syrian adolescents reported experiencing difficulties in their lives associated with remembering the demolition of their households in Syria, the death of siblings and/or parents, relatives, or neighbors, traveling under a range of threatening conditions, displacement, and bombing. Racial discrimination and violence against adolescents were reported by most primary healthcare professionals and adolescents.

### Gender and nationality differences

Based on their experiences in dealing with patients, primary healthcare professionals stated that, unlike Jordanian adolescents, Syrian adolescents felt less safe and less supported. Almost all schoolteachers reported that they observed that Jordanian adolescents are more settled, self-confident, and financially stable relative to their Syrian peers. They also reported that Syrian adolescents are more aggressive and complain more about financial constraints. This, according to their experiences, resulted in a higher percentage of Syrian adolescents dropping from school and becoming involved in the labor market. A male teacher said: “Syrian *adolescents are suffering heavily from financial constraints and limited resources, which have resulted in leaving schools and going to work to provide support for their families”* Early marriage was reported by healthcare professionals and schoolteachers as a common practice among the study population.

Based on their practices dealing with adolescents, most schoolteachers and health care professionals said that adolescents from both genders suffered so much but the female refugees were at a greater disadvantage than the males. A female physician said: “*Syrian females are suffering more than males from psychosocial problems, especially because of thoughts of being forced to early marriage*.”

### Causes of psychosocial problems among adolescents

According to Syrian parents, the presence of psychosocial problems in adolescents is linked to several factors, including the ongoing crisis in Syria, the illness or death of the head of the household (often the father), discrimination against Syrians, domestic issues such as separation and parental cruelty, as well as financial constraints. These factors have had a significant impact on the well-being of Syrian adolescents and have contributed to the development of various psychosocial concerns that they are facing.

### The impact of psychosocial problems on Syrian adolescents

During discussions, healthcare providers and schoolteachers emphasized the impact of psychosocial problems on Syrian adolescents. They widely agreed that these problems are resulting in feelings of hopelessness, violence, negativity, and hostility among adolescents. Furthermore, they noted that these issues are affecting different aspects of the lives of Syrian refugees, such as poor educational attainment, emotional and behavioral difficulties, and challenges in peer relationships. Based on their experience in managing psychosocial problems among adolescents, healthcare professionals reported that common issues they encounter include decreased appetite, lack of focus and sleep, and bed-wetting.

Parents of Syrian refugee adolescents reported that their children experienced bad effects of psychosocial problems including child labor. A Syrian mother said: “*I feel that children, especially my child, feel introverted and isolated because of the events and the conflicts that the Syrians suffered from, particularly the loss of relatives and the martyrdom of his uncle in the events. My daughters are embarrassed for not being able to complete their education and for staying at home for many years and being late for several stages in the study*.” On the other hand, Syrian adolescents reported that the problems affected their mental status and educational achievement.

### Violence against Syrian children in Jordan

Healthcare professionals, teachers, and Syrian parents emphasized that verbal abuse and physical violence are commonly observed against adolescents, which are becoming less frequent when compared to previous years. However, the health care professionals and teachers reported that the help offered by different non-governmental agencies, the Department of family protection in Jordan, and other humanitarian assistance organizations is significant. In addition, they reported that educational advisors and teachers, financial support, social activities, and services, educating the parents, and reporting cases of violence had a great role in supporting the abused adolescents in Jordan. A male professional from the health sector said: “*Increasing the awareness of the community through religious leaders and teachers is an effective method to reducing violence exerted against Syrian adolescents*.”

### Approaches that helped Syrian adolescents in Jordan

Almost all Syrian parents reported that the Jordanian government and community helped Syrian adolescents by embracing, welcoming them, and providing financial, and psychological support whereby many efforts are made to ensure that students continue their education, making recreational centers available for them, providing health services, and awareness campaigns. In addition, they mentioned that humanitarian organizations had a great role in helping their children.

### Coping mechanisms

Syrian adolescents reported that they are living with the fear of bombing and war, displacement, poverty, health problems, racial discrimination between Jordanians and Syrians, lack of sleep and concentration, thinking of early marriage (for girls), problems with stepmothers (for fathers, who have more than one wife), feelings of insecurity, poor educational attainment, psychological stress, and instability. They reported that the main coping mechanisms they use include going to schools, praying and reading the Holy Quran, listening to music, and talking to friends and engaging with them. In addition, they reported other mechanisms including seeking help from parents, focusing heavily on the study, engaging in activities such as cooking, and seeking help from teachers, family protection departments, associations, and parents. A female Syrian adolescent said: “*Before bedtime, we were reading the Quran and praying to stay alive for the next day*’’.

### Unmet psychosocial needs for Syrian adolescents

The majority of Syrian parents said that more services are still needed for adolescents, including more places for entertainment, psychosocial support and psychological counseling, medical care, job creation, and providing health insurance. According to healthcare professionals and teachers, free and specialized health services, health education, awareness campaigns, child support and protection, and psychological, financial, and social support are still needed for Syrian adolescents. Syrian adolescents highlighted the need for recreational areas, awareness campaigns, and entertainment activities.

## Discussion

This study showed that Syrian refugee adolescents in Jordan suffer from considerable levels of psychosocial problems. Many factors affecting the mental and psychosocial health of Syrian refugees relate to the traumatic events during the conflict, serious displacement experiences, resettlement, integration, and adaptation to problems of the new location (39, 40). This study reported that these problems are still affecting Syrian adolescents in Jordan after 8 years of the beginning of the Syrian conflict.

This study showed that many psychosocial problems were widespread among adolescent Syrian refugees including fear, lack of a sense of security, loneliness, isolation, aggressiveness, family disintegration, poor educational attainment, racial discrimination, and violence. Adolescent Syrian refugees were battling with problems related to the war experience and displacement, such as the destruction of their house in Syria, the killing of their parents, relatives, and neighbors, bombing, poor educational attainment, financial problems, loss of school years, and family dispersion.

The findings of this study are consistent with the findings of previous assessments and studies (39, 41). These studies showed that Syrian children and adolescents in Jordan were most affected by the conflict and displacement. School environment problems including bullying, racial discrimination, and poor educational attainment were reported as the main sources of distress in other studies (30, 31, 33). Moreover, these studies reported that social isolation, sadness, and aggressiveness symptoms were common in distressed children and adolescents (39, 41).

In Lebanon, it was reported that Syrian refugees had exerted high pressure on the health system and economy of Lebanon, causing a Lebanese Syrian crisis (16–19, 23, 27, 42). Moreover, adolescent Syrian refugees in Lebanon suffer from perceptions of insecurity due to the conflict and displacement experiences, education and study problems, and poor financial situation of families due to the lack of job opportunities and high rental rates (25, 27).

In Turkey, the increased influx of Syrian refugees into the country added a further burden to the healthcare system and caused incompetence to provide the needed healthcare services for children such as immunization programs and drugs (20, 43). Moreover, some studies in Turkey suggested the presence of different hidden mental problems and needs among Syrian refugee children living in Turkey and showed that problems of anxiety among Syrian children and violence are considerable (36, 37, 44).

In Europe, Syrian refugees suffered from not being employed because of the language barrier (45). In addition, it was reported that Syrian refugees in Europe manifested symptoms of emotional problems, such as grief, depression, anxiety, stress, anger, and cognitive problems including hopelessness, helplessness, and lack of control, also physical problems such as changes in the sleep cycle and appetite, and fatigue.

Our study showed that Syrian adolescents used different mechanisms to cope with the harsh situation of having fled from their homes such as praying and reading the Holy Quran, listening to music, talking to, and engaging with friends, focusing heavily on their studies, engaging in activities such as cooking and seeking help from parents, teachers, family protection department, associations, and parents. The coping mechanisms that had been reported by the IMC study in the same population were generally spiritual practices such as praying and individual acts such as drawing, sleeping, and going out. In Europe, on the other hand, Syrians showed high resilience and ability to cope and adapt to distressing situations despite the hardship they experienced, particularly during the early months of their presence in the hosting countries (46). These findings are different from those reported in our study.

## Conclusion

Many psychosocial problems are still affecting Syrian adolescent refugees in Jordan although many of them started living in Jordan many years ago should be adopted to provide mental health and psychosocial services including family and community support and focused non-specialized supports and services (47). There is a need to conduct more awareness campaigns on mental health and available support services for vulnerable adolescents. Gender-specific mental health support programs should be developed, taking into account the unique concerns of each group, and confidentiality should be maintained to encourage vulnerable adolescents to seek help from mental health support institutions. Regular mental health assessments should be conducted, and group therapy in community centers should be implemented. Regular assessments of schools, social and neighborhood environments should be conducted, and activities promoting interaction with community members should be organized to enhance resilience traits and perceived social support in adolescents. Central supportive groups consisting of professionals and community members should be established in all regions to help adolescents.

Addressing the financial situation of families is also critical in addressing the psychosocial problems faced by Syrian adolescents. There is a need to develop job opportunities for Syrian refugees in Jordan, decrease child labor, and provide financial support to those who have low incomes and live below the poverty line. Affordable housing is also a significant need for families, and solutions should be explored to address this issue.

## Data availability statement

The raw data supporting the conclusions of this article will be made available by the authors, without undue reservation.

## Ethics statement

The studies involving human participants were reviewed and approved by Institutional Research Committee of Jordan University of Science and Technology. Written informed consent to participate in this study was provided by all adult participants while adolescents provided informed consent through participants’ legal guardian/next of kin.

## Author contributions

TA-S conceived and designed the study, conducted research, provided research materials, and collected and organized data. YK, LH, and OA analyzed and interpreted data. AJ, MA, BA, and HA wrote the initial and final draft of the article and provided logistic support. TA-S, NA, and HA reviewed, verified, validate, and finalize the final extracted data and manuscript. All authors contributed to the article and approved the submitted version.

## Conflict of interest

The authors declare that the research was conducted in the absence of any commercial or financial relationships that could be construed as a potential conflict of interest.

## Publisher’s note

All claims expressed in this article are solely those of the authors and do not necessarily represent those of their affiliated organizations, or those of the publisher, the editors and the reviewers. Any product that may be evaluated in this article, or claim that may be made by its manufacturer, is not guaranteed or endorsed by the publisher.
