# Supplementary material for: Identifying psychosocial problems, needs, and coping mechanisms of adolescent Syrian refugees in Jordan
Source: Front Psychiatry. 2023 Jun 22;14:1184098. doi: 10.3389/fpsyt.2023.1184098 (PMC10325618; doi:10.3389/fpsyt.2023.1184098)
Supplement: Supplementary file 1 [file Table_1.DOCX]

**Supplementary 1.** A qualitative interview guide with key and individual informants in the study in both languages, Arabic (original) and English (translated).

| Primary healthcare providers and Schoolteachers | |
| --- | --- |
| 1. What are the most important psychological and social problems faced by Syrian children aged 12 to 17 in Jordan? | 1. ما هي اهم المشاكل النفسية والاجتماعية التي يواجهها الاطفال السوريون من عمر 12 حتي 17 سنة في الأردن؟ |
| 1. How do these problems differ from those faced by Jordanian children aged 12 to 17 in Jordan? How? | 1. هل تختلف هذه المشاكل عن التي يواجهها الاطفال الاردنيون من عمر 12 حتى 17 سنة في الأردن؟ كيف؟ |
| 1. Which groups of Syrian children suffer the most, males or females? And what about the other group? | 1. أي فئات الأطفال السوريين تعاني أكثر، الذكور أم الاناث؟ وماذا عن الفئة الأخرى؟ |
| 1. How do these problems affect children? | 1. كيف تؤثر هذه المشاكل على الاطفال؟ |
| 1. What is the extent of violence and bullying experienced by Syrian children in Jordan? | 1. ما مدى العنف والتنمر الذي يواجهه الاطفال السوريون قي الاردن؟ |
| 1. What are the types of violence practiced against Syrian children? | 1. ما هي انواع العنف الممارس ضد الاطفال السوريين؟ |
| 1. How can these problems be overcome for both Jordanian and Syrian children? | 1. كيف يمكن التغلب على هذه المشاكل عند الاطفال الأردنيين والسوريين؟ |
| 1. What health services or social and psychological support should be provided to Syrian children? | 1. ما هي الخدمات الصحية او الدعم الاجتماعي والنفسي الذي يجب تقديمه للأطفال السوريين؟ |
| 1. How can the community, institutions, and knowledge of Syrian children who are victims of violence, whether from their own families or the surrounding environment, help? | 1. كيف يساعد المجتمع من مؤسسات ومعارف الاطفال السوريين المعنفين سواء كان مصدر هذا العنف من الاسرة نفسها او من البيئة المحيطة؟ |
| Syrian Parents | |
| 1. What are the most important psychological and social problems faced by your children aged 12 to 17 in Jordan? | 1. ما هي اهم المشاكل النفسية والاجتماعية التي يواجهها اطفالكم من عمر 12 حتي 17 سنة في الأردن؟ |
| 1. What is the cause and source of these problems in your children? | 1. ما هو سبب ومصدر هذه المشاكل لدى اطفالكم؟ |
| 1. How do these problems affect children? | 1. كيف تؤثر هذه المشاكل على الاطفال؟ |
| 1. What are the types of violence practiced against your children? | 1. ما هي انواع العنف الممارس ضد اطفالكم؟ |
| 1. What are the biggest sources of tension between Syrian refugees and the Jordanian host community? | 1. ما هي أكبر مصادر التوتر بين اللاجئين السوريين والمجتمع الاردني المضيف؟ |
| 1. How can these problems be overcome for Syrian children? | 1. كيف يمكن التغلب على هذه المشاكل عند الاطفال السوريين؟ |
| 1. How did the community help during and after the war? | 1. كيف ساعد المجتمع الاحداث في المحنة قبل وبعد الحرب؟ |
| 1. What more can be done to help children suffering from the crisis? | 1. ما الذي يمكن القيام به أكثر لمساعدة الاحداث الذين يعانون من المحنة؟ |
| 1. What are the problems of parents who have children aged 12-17? | 1. ما هي مشاكل الآباء والأمهات الذين لديهم أحداث تتراوح أعمارهم بين 12-17سنة؟ |
| 1. Are there any organizations that support you in supporting your children who are facing problems? | 1. هل هناك جهات تساندكم في الدعم لأطفالكم الذين يعانون من المشاكل؟ |
| 1. What would support Syrian parents to take care of their children? | 1. ما الذي يمكن أن يساعد الاباء والامهات السوريين في رعاية اطفالهم؟ |
| Syrian adolescents 12-17 years old | |
| 1. What types of problems have you faced due to the war and living in Jordan? | 1. ما هي انواع المشاكل التي تعرضت لها بسبب الحرب وبسبب العيش داخل الاردن؟ |
| 1. Can you speak more about each problem you have faced? | 1. هل يمكن التحدث أكثر عن كل مشكلة من هذه المشاكل؟ |
| 1. Which of these problems is the most important and impactful, second, and third? | 1. اي من هذه المشاكل هي الأكثر أهمية والأكثر تأثيراً فالثانية فالثالثة؟ |
| 1. How does each problem affect your daily life? | 1. كيف تؤثر كل مشكلة من هذه المشاكل على حياتك اليومية؟ |
| 1. What methods do you use to overcome these problems? | 1. ما الطرق التي تستخدمها للتغلب على هذه المشاكل؟ |
