# Supplementary material for: Identifying psychosocial problems, needs, and coping mechanisms of adolescent Syrian refugees in Jordan
Source: Front Psychiatry. 2023 Jun 22;14:1184098. doi: 10.3389/fpsyt.2023.1184098 (PMC10325618; doi:10.3389/fpsyt.2023.1184098)
Supplement: Supplementary file 2 [file Table_2.DOCX]

**Supplementary 2.** Qualitative Assessment Outputs

**Health sector**

**All regions**

| **Trending**  **Ramtha** | **Trending**  **Sahab** | **Trending**  **Zarqa** | **Trending**  **Mafraq** | **Q**  **No** |
| --- | --- | --- | --- | --- |
| Family disintegration, homelessness, poverty. | Violence, nervousness, bed-wetting, fear. | Post-war trauma, fear, isolation, expatriation, emotional emptiness, loss of security, shyness, illiteracy. | Psychological stress, discrimination between the Jordanian and Syrian, poverty, war, changes in education circumstances, isolation. | 1 |
| Yes, there is a difference. The Jordanian feels safe while the Syrian is suffering from homelessness and a bad financial situation. | Yes, there is a difference. Jordanians suffer less than Syrians. | The Jordanian is looking for secondary requirements because he feels safe and not like the Syrians. | Awareness and openness among the Syrian children are more than that within the Jordanian children as a result of what they have experienced, the Jordanian child feels more safe and supported than the Syrian one. | 2 |
| Males. | Females. | Females. | Females. | 3 |
| It affects the mental state and causes isolation, loss of hope, and behavioral perversion. | It affects educational attainment, behavior, psychological status, and social relationships. | Aggressiveness, weakness, fear of claiming rights, the Syrian can be exploited materially. | It leads to psychological stress. | 4 |
| Somewhat too many. | There is racial discrimination. Violence in general is little | There are some cases of bullying and violence. | There is no violence or bullying, or it is rare if there is any. | 5 |
| Physical, psychological, and sexual violence. | Verbal, and physical violence. | Physical, verbal, and psychological violence. | Psychological violence. | 6 |
| Syrian and Jordanian integration, provision of child support and protection institutions. | Syrian and Jordanian integration workshops, awareness campaigns and educational courses. | Awareness campaigns and lectures, psychotherapy. | It is the role of families to guide and support both Syrian and Jordanian children. Also, by conducting lectures and awareness campaigns at schools and other places, and financial support. | 7 |
| Providing special centers and associations to deal with cases of violence and to study social conditions. Also, providing psychologists. | Psychological and social support through courses by specialists in psychology and sociology, and providing treatment. | Health, psychological, and financial support through relevant associations and institutions. | Free and specialized health services, health education, safe social environment. | 8 |
| Reporting cases of violence, and awareness campaigns. | Activating the role of the Family Protection Department and providing a liaison officer for cases of violence. Also, educating the parents. | Awareness campaigns, financial support, and more social activities and services. | Family protection department, humanitarian organizations, and teachers. | 9 |

**Education sector**

**All regions**

| **Trending**  **Ramtha** | **Trending**  **Sahab** | **Trending**  **Zarqa** | **Trending**  **Mafraq** | **Q**  **No** |
| --- | --- | --- | --- | --- |
| Poverty, loss of parents, lack of focus on study. | Fear, instability, not coping with the new environment, losing parents or living away from them with relatives and acquaintances, family dispersion, loneliness, and isolation. | Fear, isolation, depression, aggressiveness, constant crying, violence, lying, and family problems. | Lack of education, early marriage, fear, depression, customs, and traditions. | 1 |
| Yes, there is a difference, the Jordanian is stable and self-confident. His financial situation is better and his living and family situation is also better. | Yes, there is a difference. Jordanians have not been subjected to war problems like Syrians, but they have been affected by family problems. Family disintegration, and the social relationships problems have the biggest effect on Jordanians, but the Syrians are affected by the war environment. | Problems vary, for Jordanians most of the problems are family problems, as well as depression, for Syrians, the problems are more severe, including fear and stubbornness. | The Syrian child is more aggressive than the Jordanian, the Jordanian child has more supported life, Syrian child labor. | 2 |
| Females. | Both of them. | Females. | Females. | 3 |
| Weak academic achievement, violence, negativity, and hostility | It affects the educational attainment and the relationship of the child to others and his or her behavior. | Impact on health, psychological, educational, social, and emotional life. | It affects different aspects of their life, their conduct, education, and psychological state. | 4 |
| Somewhat. | Racial discrimination between Jordanian and Syrian causes some problems. | Exposure to violence and humiliation due to race as well as from parents due to stress and poverty. | Syrian refugee children are influenced by violence and offense. | 5 |
| Verbal and physical violence. | Verbal and physical violence. | Physical and verbal violence. | Verbal and physical child abuse. | 6 |
| Material support, awareness raising, education, counseling, promotion, and motivation. | Integration of Jordanians and Syrians through awareness campaigns, workshops, and initiatives. | Psychological and educational guidance, seeking help from security agencies, and constant communication with parents. | Awareness campaigns for both children and parents, health care services. | 7 |
| Vaccines, periodic examination, awareness, recreational activities. | Awareness campaigns, psychological support, health education, entertainment activities, and health insurance. | Psychological support through psychologists, providing more health services, recreational areas, and financial and material support. | Health education, and financial support for Syrian refugees by initiatives. | 8 |
| Educational courses, reporting cases of violence, and financial support. | Seeking help from the family protection department, awareness campaigns of parents, and constant contact with them. | Activating the role of security agencies, providing guidance, psychological and educational counseling. | By the help of educational advisors. | 9 |

**Syrian parents**

**All regions**

| **Trending**  **Ramtha** | **Trending**  **Sahab** | **Trending**  **Zarqa** | **Trending**  **Mafraq** | **Q**  **No** |
| --- | --- | --- | --- | --- |
| Fear of wars and high voices. | Fear, breakdown, caution from Jordanian society, sensitivity, depression. | Grief, crying, introversion, solitude, study problems, aggressiveness, and rage. | Fear, isolation, psychological stress. | 1 |
| The war. | As a result of the war. | Father's illness, discrimination between Syrian and Jordanian, family problems such as separation and cruelty of parents, bad material situation, fear, and technology. | Family problems. | 2 |
| Lack of educational attainment. | Negative effect on educational attainment. | It affects educational attainment and Leads to aggressiveness, violence, fear, rebellion, isolation, and crying. | Fear, Syrian child labor, aggression. | 3 |
| Physical and verbal. | Verbal violence. | There is no violence. | There is no violence. | 4 |
| Racial discrimination,  competition for jobs. | Differences in views and opinions, competition for jobs, racism, and bias. | Racial discrimination, and competition for sustenance. | There is no tension. | 5 |
| integration between Syrians and Jordanians. | Courses and activities in support of the Syrian-Jordanian integration. | Awareness, education, and material support. | The role of family education, psychosocial support of children. | 6 |
| Associations, free education. | Before the war, everything was better. After the war, providing education, awareness campaigns, compassion to help them, the insurance, and health support for them is what they require. | Help through humanitarian organizations, psychological counseling, helping students to complete their studies, and providing recreational centers. | Embrace of a Syrian refugee by Jordanians, and financial support. | 7 |
| Job creation, training, providing psychotherapy and safety. | Increasing care of children by parents, providing recreational facilities for children, improving economic situation and providing health insurance. | Psychological counseling, medical care, financial support, job creation. | Financial support, and providing places for entertainment. | 8 |
| Financial problems, children's rebellion. | Stubbornness, determination, defiance, conflicts between parents and children, rebellion, bullying, poor financial status, and inability to meet the needs of children. | Financial problems and failure to meet the needs of children, family problems such as separation and remarriage, and study problems such as refusal to study. | The need for financial and psychological support. | 9 |
| By associations. | UNICEF. | There are no agencies to support children. | There are no organizations for child support. | 10 |
| Job creation. | Improving economic conditions through providing centers, schools, and kindergartens for Syrians. | Educational and psychological counseling, financial support. | Financial and psychological child support, and awareness campaigns for parents. | 11 |

**Syrian adolescents**

**All regions**

| **Trending**  **Ramtha** | **Trending**  **Sahab** | **Trending**  **Zarqa** | **Trending**  **Mafraq** | **Q**  **No** |
| --- | --- | --- | --- | --- |
| Loss of family, poverty, war. | War, fear, displacement, loss of family, racial prejudices, lack of sense of security, financial loss, loss of school years, family dispersion, changes in work circumstances, and increased pressure from parents. | Fear of voices, solitude, racial discrimination, poor educational attainment, alienation, death of relatives, problems with friends and colleagues, violence, and tight living. | Destruction of the house in Syria, killing parents, relatives, and neighbors, displacement, bombing, fear, and anxiety. | 1 |
| Loss of family, and instability. | Because of war, fear, displacement, alienation, feelings of insecurity, poor educational attainment, psychological stress, loss of family, and homelessness. | A narrow house, lack of sleep and concentration, death of parents, thinking of early marriage, problems with stepmother, Immigration Walking Tour, racial discrimination. | Fear of shelling, the martyrdom of the father, displacement, frustration, poverty, health problems, racial discrimination between Jordanians and Syrians. | 2 |
| Firstly, stepmother,  loss of family, alienation, poverty, sister injury, and school mobility. | Firstly, migration, fear, and war. | Firstly, expatriation and death of parents. | Firstly, the death of the father or mother and illness, and secondly, the displacement. | 3 |
| Feeling isolated, and lacking the desire to communicate with others. | It affects the mental state and educational achievement and causes isolation from society and loneliness. | Introvert, not eating enough, lack of concentration and sleep. | The loss of the father greatly affects our life, misunderstanding with Jordanian colleagues, sadness, fear, frustration, and depression. | 4 |
| Seeking help from teachers, the family protection department, associations, and parents. | By engaging with friends, focusing heavily on the study, and engaging in activities such as cooking. | Trying to solve problems and face them, seeking help from parents. | Continuing education, making dreams come true, Prayers, patience, listening to music, talking to friends, Quran. | 5 |
